# Heme Oxygenase-1 Accelerates Cutaneous Wound Healing in Mice

**DOI:** 10.1371/journal.pone.0005803

**Published:** 2009-06-04

**Authors:** Anna Grochot-Przeczek, Radoslaw Lach, Jacek Mis, Klaudia Skrzypek, Malgorzata Gozdecka, Patrycja Sroczynska, Milena Dubiel, Andrzej Rutkowski, Magdalena Kozakowska, Anna Zagorska, Jacek Walczynski, Halina Was, Jerzy Kotlinowski, Justyna Drukala, Krzysztof Kurowski, Claudine Kieda, Yann Herault, Jozef Dulak, Alicja Jozkowicz

**Affiliations:** 1 Department of Medical Biotechnology, Faculty of Biochemistry, Biophysics and Biotechnology, Jagiellonian University, Krakow, Poland; 2 Department of Cell Biology, Faculty of Biochemistry, Biophysics and Biotechnology, Jagiellonian University, Krakow, Poland; 3 R&D Division, Adamed Ltd., Pienkow, Poland; 4 Centre for Molecular Biophysics, CNRS, Orleans, France; 5 Centre for Transgenic Animals, CNRS, Orleans, France; Ohio State University Medical Center, United States of America

## Abstract

Heme oxygenase-1 (HO-1), a cytoprotective, pro-angiogenic and anti-inflammatory enzyme, is strongly induced in injured tissues. Our aim was to clarify its role in cutaneous wound healing. In wild type mice, maximal expression of HO-1 in the skin was observed on the 2^nd^ and 3^rd^ days after wounding. Inhibition of HO-1 by tin protoporphyrin-IX resulted in retardation of wound closure. Healing was also delayed in HO-1 deficient mice, where lack of HO-1 could lead to complete suppression of reepithelialization and to formation of extensive skin lesions, accompanied by impaired neovascularization. Experiments performed in transgenic mice bearing HO-1 under control of keratin 14 promoter showed that increased level of HO-1 in keratinocytes is enough to improve the neovascularization and hasten the closure of wounds. Importantly, induction of HO-1 in wounded skin was relatively weak and delayed in diabetic (db/db) mice, in which also angiogenesis and wound closure were impaired. In such animals local delivery of HO-1 transgene using adenoviral vectors accelerated the wound healing and increased the vascularization. In summary, induction of HO-1 is necessary for efficient wound closure and neovascularization. Impaired wound healing in diabetic mice may be associated with delayed HO-1 upregulation and can be improved by HO-1 gene transfer.

## Introduction

The repair of wound is a complex process engaging activity of various cell types, tightly orchestrated by specific cytokines. The molecular and cellular machinery, which is switched on in response to destructive stimuli, is very similar in case of burn, cutaneous wounds, myocardial infarction, spinal-cord damage, and visceral injuries [1]. Noteworthy, chronic nonhealing wounds, leading very often to ulceration, necrosis and amputation, are one of the severe consequences of diabetes, which result in significant morbidity. Especially in this case, the new avenues for therapeutic approaches of deep skin injuries are urgently needed.

In mammals, skin wound healing occurs in three overlapping phases: inflammation, granulation tissue formation and remodeling [2]. Briefly, the injury causes immediate blood vessel constriction and activation of coagulation cascade. When the blood clot is formed, neutrophils and monocytes enter the wound site. Then, keratinocytes and fibroblasts are stimulated to proliferate and migrate over the provisional matrix, so the granulation tissue formation begins. During the final phase of wound healing the granulation tissue is replaced with an acellular scar, when myofibroblastic and vascular cells in the wound undergo apoptosis [3].

Vascularization of wound, achieved both by angiogenesis (formation of blood vessels from preexisting ones) and vasculogenesis (formation of blood vessels from endothelial progenitor cells), is necessary for quick and effective tissue repair. It has been shown that endostatin, an angiogenesis inhibitor, delayed wound healing through attenuation of granulation tissue formation and vascularization in mice [4], and impaired maturation of blood vessels during the restoration of skin integrity [5], although reepithelialization of wound was not affected [4], [5]. Moreover, neutralizing antibodies against vascular endothelial growth factor (VEGF), the major angiogenic and vasculogenic mediator, inhibited formation of granulation tissue and decreased number of blood vessels [6], whereas VEGF overexpression by *ex vivo* expanded keratinocyte cultures promoted matrix formation, angiogenesis, and healing in porcine full-thickness wounds [7]. Also diabetic wound healing was accelerated in response to VEGF via increased angiogenesis and recruiting bone marrow-derived cells [8]. Finally, transplantation of endothelial progenitor cells (EPCs) improved dermal wound healing, showing the potency of blood vessel-directed cell therapy [9].

One of the important molecular mediators of new blood vessel formation is heme oxygenase-1 (HO-1). This cytoprotective enzyme, strongly induced by pro-oxidants and inflammatory agents, degrades heme to three compounds: CO, free iron and biliverdin, which is subsequently reduced to bilirubin by biliverdin reductase [10]. Previous studies have revealed the significance of HO-1 in regulation of synthesis and activity of VEGF [11]–[14] and its role in angiogenesis [15], [16]. Recently we have indicated an importance of HO-1 in stromal cell derived factor-1 (SDF-1)-dependent neovascularization, showing delayed wound closure and poor vascularity of wounded skin in 3-month old HO-1 knockout mice [17].

It has been shown that HO-1 is rapidly induced in the wounded tissues [18], [19]. Keeping in mind its pro-angiogenic, anti-inflammatory and cytoprotective properties, as well as its influence on cell proliferation and migration [20], one can suppose that expression of HO-1 may play a role in the process of wound healing. The aim of our study was to verify this hypothesis. We evidenced the significance of HO-1 in skin repair, and demonstrated that delayed HO-1 induction may contribute to the impairment of wound healing in diabetes. Accordingly, we showed that HO-1 gene transfer may facilitate wound closure and neovascularization in diabetic mice.

## Results

### Effect of SnPPIX on wound closure

**Figure 1:**
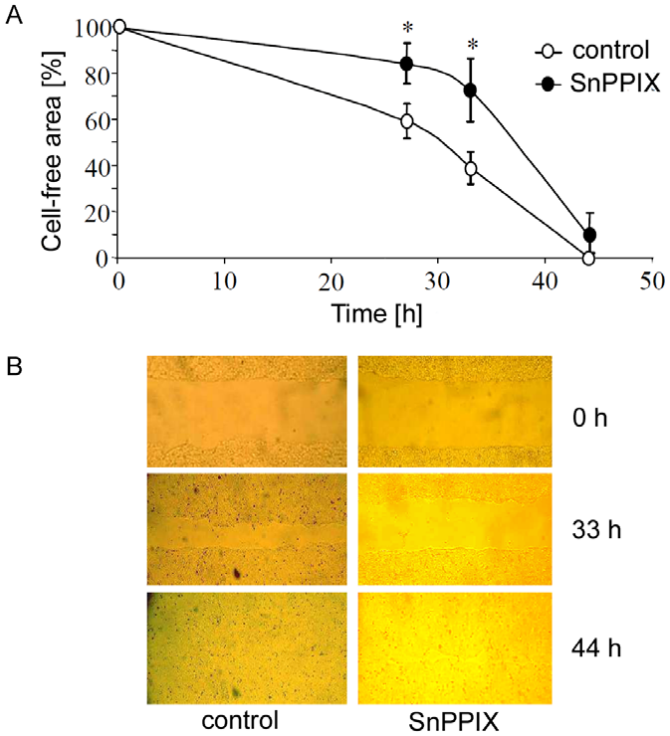
Effect of SnPPIX (10 µmol/L) on time of gap closure by HaCaT cells cultured *in vitro* in the presence of hydroxyurea (10 mmol/L). Scratch assay. A – Quantitative data. Each point represents mean±SD of 3 experiments done in duplicates. * P<0.05 in comparison to control. B – representative pictures. Scale bar = 200 µm.

First experiments were performed *in vitro* on human keratinocyte cell line HaCaT, using a scratch assay model. Incubation of cells with tin protoporphyrin (SnPPIX, a competitive inhibitor of heme oxygenases, 10 µmol/mL) significantly slowed down the closure of gap created on the confluent cell monolayer (Fig. 1A,B). Most likely this resulted from reduction of keratinocyte migration, not proliferation, as all experiments were performed in the presence of 10 mM hydroxyurea, a cell cycle blocker.

**Figure 2:**
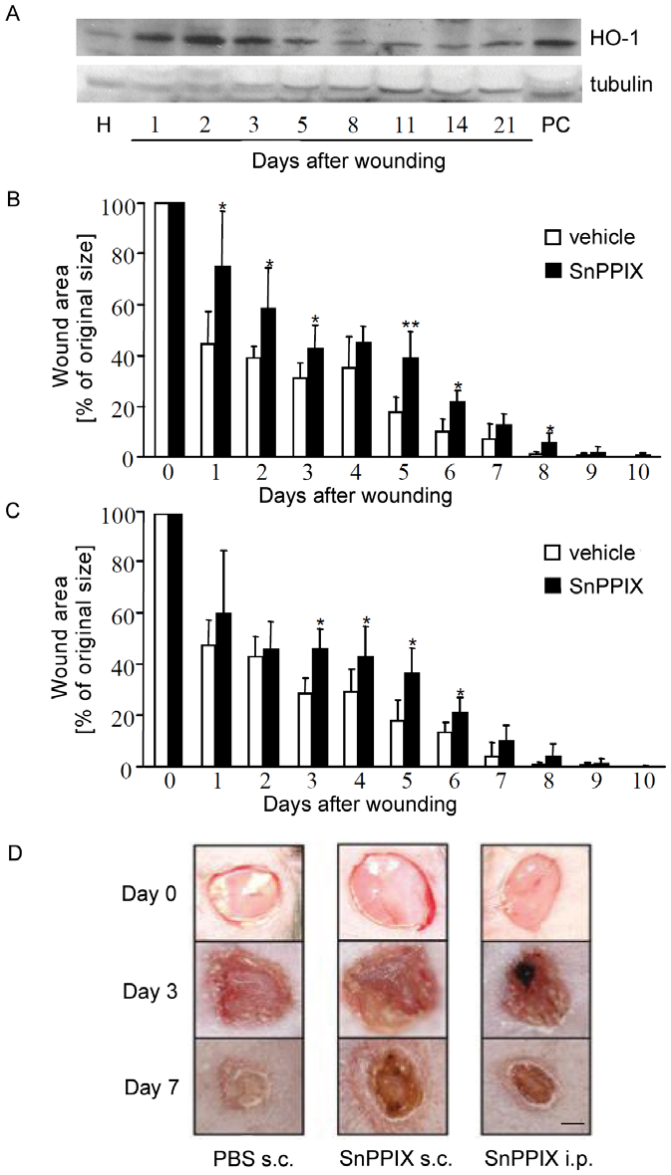
A - Expression of HO-1 protein in healthy and wounded skin. H - healthy skin, PC - positive control (HaCaT cells stimulated with 10 µmol/L of hemin for 24 h). Western blot analysis. Tubulin was used as a housekeeping gene to control the protein loading. One of 3 similar blots. B - Effect of SnPPIX (45 µmol/kg of body weight) injected subcutaneously (once a day for 10 days) on wound closure in C57BL mice. C – Effect of SnPPIX (45 µmol/kg of body weight), injected intraperitoneally (once a day for 10 days) on wound closure in C57BL mice. Each bar represents mean+SD; N = 10 animals per group. * P<0.05, ** P<0.01 in comparison to control, vehicle injected animals. D – representative pictures taken immediately after wounding, and on the 3^rd^ and 7^th^ days. Scale bar = 1 mm.

Then we investigated the wound healing process *in vivo*, in 3-month old C57BL wild type mice. Two circular wounds (4 mm in diameter) on the dorsum of each animal were created using biopsy punch. In the first step we checked the effect of wounding on HO-1 expression. By means of western-blotting we investigated the level of HO-1 protein in the tissue lysates prepared from healthy and wounded skins. As shown in Fig. 2A, expression of HO-1 in the healthy tissue was relatively low. Upon injury it was rapidly upregulated and remained very high on day 1^st^, 2^nd^, and 3^rd^, then gradually decreased.

In the second step we examined the effect of SnPPIX on wound healing. To this end, immediately after wounding, mice were divided into four groups and injected every day for 10 consecutive days either intraperitoneally (*i.p.*) or subcutaneously (*s.c.*) with SnPPIX or with vehicle. The animals were photographed every day to measure the wound surface. Analysis of images revealed that mice treated *s.c.* with SnPPIX showed a delayed wound closure compared to mice injected with the vehicle, which was evident throughout the whole process of healing, beginning from day 1^st^ (Fig. 2B,D). The first fully closed wounds in control mice were found on the 7^th^ day after wounding, whereas in SnPPIX-treated group they appeared earliest on day 8^th^. In case of *i.p.* injected animals, the delay did not become apparent until the 3^rd^ day after injury, which might be associated with less effective penetration of the inhibitor to the wounded tissue than after local injection. However, similarly as in the *s.c.* treated individuals, the delayed wound healing was clearly visible (Fig. 2C,D). Again, in the vehicle injected mice the fully closed wounds appeared first on the 7^th^ day after wounding, whereas in the SnPPIX treated group on the 8^th^ day. Thus, *s.c.* and *i.p.* injections of SnPPIX result in impaired wound closure in mice, which may suggest that HO-1 plays a role in the process of wound healing.

### Effect of HO-1 deficiency on wound healing

SnPPIX is commonly used to suppress HO-1 activity in cell culture and animal experiments. However, it exerts also a variety of non-specific, HO-1 independent effects [21], [22]. Therefore, to confirm the importance of HO-1, we investigated the wound healing process in C57BLxFVB mice of normal level of HO-1 (HO-1^+/+^) and mice lacking one (HO-1^+/−^) or both (HO-1^−/−^) HO-1 alleles.

**Figure 3:**
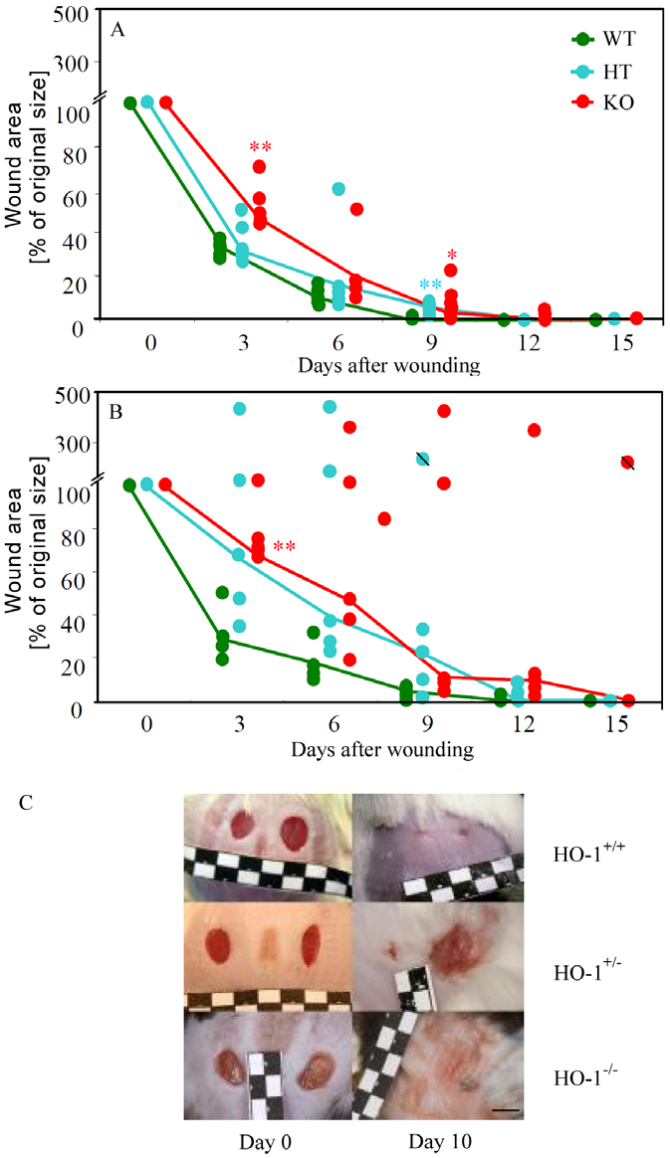
Closure of cuteneous wounds in the HO-1^+/+^ (WT), HO-1^+/−^ (HT), or HO-1^−/−^ (KO) C57BLxFVB mice. A – 3-month old animals. B – 6-month old animals. Each point represents individual animal (N = 4–5), lines connect the median values. Crossed points represent animals subjected to euthanasia. * P<0.05, ** P<0.01 in comparison to WT. C – representative pictures showing the wounds in 6-month old animals immediately after wounding and on day 10^th^. Scale bar = 5 mm.

As we have already noticed in the earlier paper [17], lack of HO-1 was associated with significantly delayed wound closure in the 3-month old HO-1^−/−^ mice compared to the wild type animals of the same age (Fig. 3A). Noteworthy, the most pronounced differences were observed during the first phases of healing, between 1^st^ and 6^th^ day after injury, at the time of the strongest induction of HO-1 in the wounded skin (Fig. 2A). The rate of wound healing in the heterezygous (HO-1^+/−^) animals, was similar to that in HO-1^+/+^ mice (Fig. 3A).

Effect of HO-1 deficiency on wound healing in the older, 6-month old animals, was remarkable. Time required for wound closure in the wild type mice did not differ significantly compared to the younger ones. However, lack of even one functional HO-1 allele resulted in a potent delay in wound healing (Fig. 3B). In some individuals (both HO-1^+/−^ and HO-1^−/−^) wounds did not cure at all and their surface increased with time forming large unhealing lesions (Fig. 3C). Such animals had to be subjected to euthanasia before the planned end of experiment. Immunohistochemical staining of CD31-positive endothelial cells, carried out on the day 17^th^ after injury, demonstrated that number of blood vessels was lower in nonhealing than in healing skins of HO-1 deficient individuals (9.76±1.64 *versus* 11.84±1.66 of vessels per surface unit, P = 0.035). Necrotic areas were also visible within the wounds. However, the mechanism behind reduced vascularization is not clear, as we did not observe any significant differences in concentration of VEGF, SDF-1 or tumor necrosis factor-α (TNFα) in the tissue lysates prepared from the healing and nonhealing wounds (data not shown).

### Effect of SnPPIX and HO-1 deficiency on production of inflammatory mediators

Since restoration of tissue integrity and function is strongly dependent on inflammation, we compared the influence of subcutaneous injection of SnPPIX and HO-1 deficiency on a profile of cytokines in the blood serum of mice on the 3^rd^ day post-wounding, using a protein macroarrays (RayBiotech).

We found that SnPPIX strongly decreased concentrations of many inflammatory mediators in the blood of wounded mice (Tab. 1). Some of those effects were possibly HO-1 dependent, as similar downregulations were observed in HO-1^+/−^ and HO-1^−/−^ animals. Namely, the coherent results were obtained for soluble CD40 antigen (sCD40), granulocyte-macrophage colony stimulating factor (GM-CSF), interferon-γ (IFNγ), interleukin-2 (IL-2), IL-17, and KC (murine ortholog of IL-8). One can suppose that reduction of some of these mediators may potentially contribute to impairment of wound healing in response to pharmacological or genetic inhibition of HO-1.

**Table 1:** List of genes differently expressed in animals with pharmacologically or genetically inhibited HO-1, 3 days after wounding.

Gene	C57Bl WT+PBS	C57Bl +SnPPIX	C57BlxFVB WT	C57BlxFVB HT	C57BlxFVB KO
** CD40**	+	−	+	−	−
** GM-CSF**	+	−	+	−	−
** IFNγ**	+	−	+	−	−
** IL-2**	+	−	+	−	−
** IL-17**	+	−	+	−	−
** KC**	+	−	+	−	−
** VEGF**	+	+	+	−	−
**Lymphotactin**	+	+	+	−	−
** CCL-24**	+	+	+	+	−
** Leptin**	+	+	+	+	−
** CCL-27**	+	+	−	+	+
** CXCL-16**	+	+	−	+	+
** CXCL-13**	+	+	−	−	+
** IL-3**	+	+	−	−	+
** CD30T**	+	−	−	+	+
** CXCL-10**	+	−	−	+	+
** IL-13**	+	−	−	+	+
** IL-10**	+	−	−	−	+
** IL-12 p70**	+	−	−	−	+

Presence of proteins in the serum of mice was analyzed using RayBiotech arrays followed by densitometric measurements. Data are shown as semiquantitative estimations of signals: + indicates OD>0.1 of value measured for positive control.

In case of VEGF we observed decreased concentration in HO-1^+/−^ and HO-1^−/−^ mice, but not in animals treated with SnPPIX, where inhibition of HO-1 might be too weak. Tendency for reduced production of VEGF in HO-1 deficient mice was confirmed by ELISA performed in cell lysates prepared from the wounded skins 3 days after injury, where VEGF concentration in comparison to HO-1^+/+^ animals was decreased to 62.4±41.0% (P = 0.075) and to 74.9±16.2% (P = 0.071) for HO-1^+/−^ and HO-1^−/−^, respectively. Interestingly, we also observed significant decrease in amount of SDF-1, which reached 44.6±21.9% (P = 0.005) in HO-1^+/−^ and 35.6±18.0% (P = 0.002) in HO-1^−/−^ mice, in comparison to values measured in wild type individuals.

Finally, several effects of SnPPIX were clearly HO-1 independent, being opposite to the results of HO-1 deficiency, as demonstrated for soluble CD30T antigen, chemokine CXCL-10, IL-10, IL-12 or IL-13. Keeping in mind that both pharmacological and genetic HO-1 inhibition impairs wound healing, we suppose that those mediators are not involved in the process studied.

### Effect of skin-specific HO-1 overexpression on wound healing

**Figure 4:**
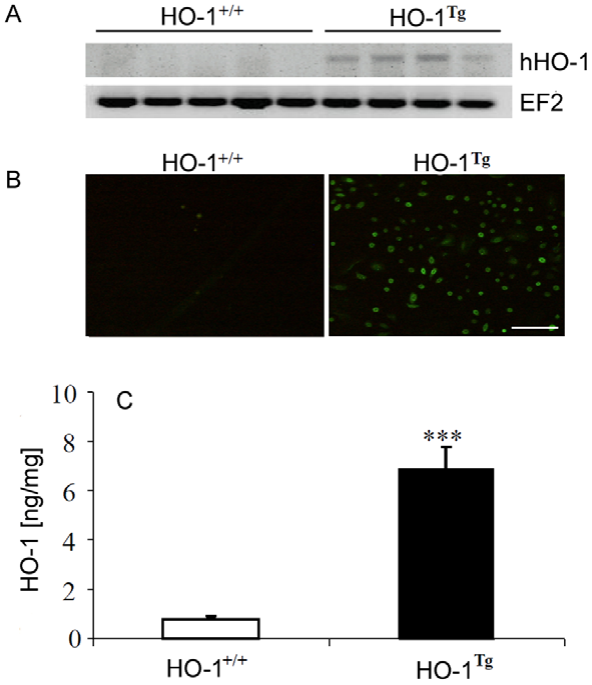
HO-1 overexpression in murine keratinocytes. A – Expression of human HO-1 mRNA in the skin of HO-1^+/+^ and transgenic HO-1^Tg^ mice C57BL mice. Electrophoresis of RT-PCR products (2% agarose gel). EF2 was used as a housekeeping gene. One of 4 similar analyses. B – Representative pictures of immunocytofluorescent staining for HO-1 in primary murine keratinocytes isolated from newborns and cultured *in vitro*. Scale bar = 100 µm. C – Concentration of HO-1 in lysates of primary murine keratinocytes isolated from newborns and cultured in vitro. ELISA. Each bar represents mean+SD of 6 measurements. *** P<0.001 in comparison to HO-1^+/+^.

We decided to investigate whether local overexpression of HO-1 may facilitate wound healing. Therefore, we created the transgenic mice (HO-1^Tg^) bearing human HO-1 gene under control of the human keratin 14 promoter to overexpress HO-1 specifically in keratinocytes, in the epidermal layer of the skin. Analysis of the primary keratinocytes isolated from such mice and cultured *in vitro* evidenced the upregulated HO-1 expression both at mRNA level, as shown by RT-PCR (Fig. 4A), and at protein level, as visualized by immunohistochemical staining (Fig. 4B) and quantified by ELISA (Fig. 4C). Such overexpression was not observed in other cell types analyzed (data not shown).

**Figure 5:**
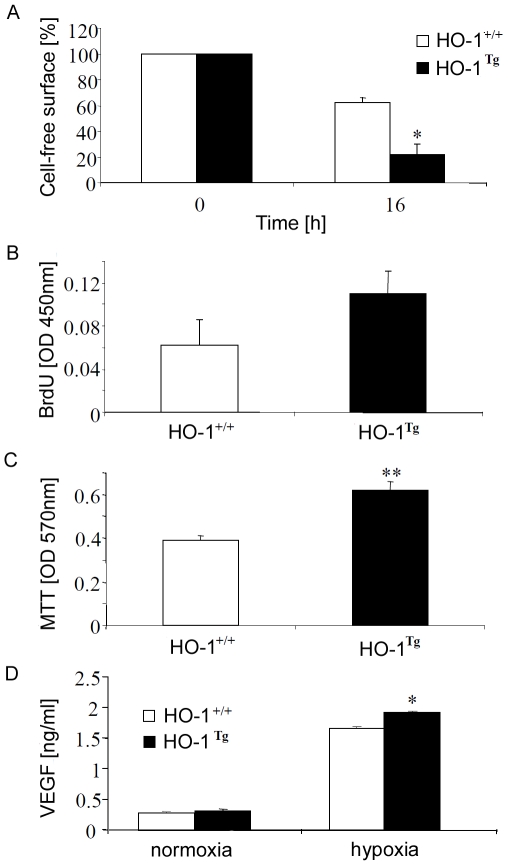
Activity of primary murine keratinocytes isolated from HO-1^+/+^ and HO-1^Tg^ newborns and cultured *in vitro.* A – Migration of cells measured by time of gap closure in the presence of hydroxyurea (10 mmol/L). Scratch assay. B – Spontaneous proliferation of cells cultured for 48 h. BrdU incorporation assay. C – Viability of cells cultured in hypoxia (1% O_2_) for 24 h. MTT reduction assay. D – Concentration of VEGF in media harvested from cell cultures after a 24 h incubation. Each bar represents mean+SD of 3–5 experiments. * P<0.05, ** P<0.01 in comparison to HO-1^+/+^.

Interestingly, the augmented expression of HO-1 accelerated the closure of scratch in a monolayer of primary transgenic keratinocytes, as demonstrated by *in vitro* wound healing assay (Fig. 5A). This indicates that HO-1 improves migration of keratinocytes. Cells isolated from transgenic mice displayed also a tendency to increased proliferation, however this did not reach statistical significance (Fig. 5B). Finally, HO-1 overexpression improved viability of cells kept for 24 h under hypoxic conditions (1% O_2_) (Fig. 5C), and slightly increased production of VEGF induced by hypoxia (Fig. 5D).

**Figure 6:**
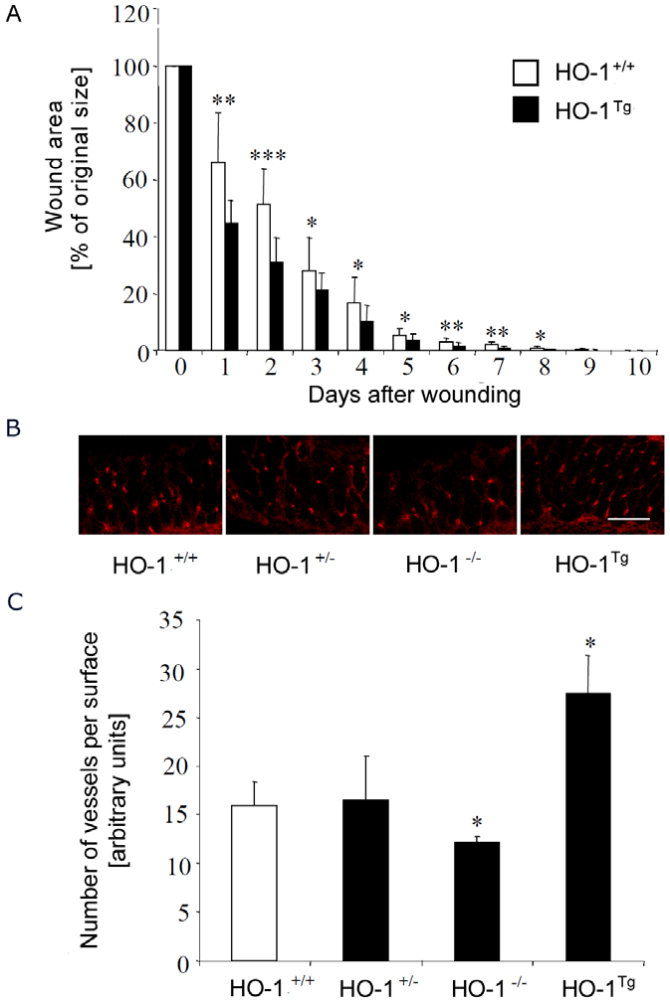
A – Closure of cutaneous wounds in the HO-1^+/+^ wild type and HO-1^Tg^ mice. Each bar represents mean+SD. N = 10 animals per group. * P<0.05, ** P<0.01, *** P<0.001 in comparison to HO-1^+/+^ mice. B – Representative pictures demonstrating CD31 staining of endothelial cells in the wounded skin (3 days after wounding) in the 3-month old mice of different genotypes. Scale bar = 100 µm. C – Number of vessels in wounded skin (3 days after wounding, CD31 staining) in the 3-month old mice of different genotypes. Each bar represents analysis of samples from 5–8 animals. Data are presented as mean+SD. * P<0.05 in comparison to HO-1^+/+^ animals.

Wound healing experiments performed *in vivo* on HO-1^Tg^ mice showed that increased expression of HO-1 in keratinocytes is enough to accelerate significantly the wound closure throughout the whole process of healing, starting from day 1^st^ (Fig. 6A).

### Effect of HO-1 expression on vascularization of wounds

Vascularization of wounds was assessed in 3-month old mice of different genotypes by immunohistochemical staining of endothelial cells using anti-CD31 antibodies. It turned out that density of blood vessels, measured three days after injury, reflected very well the rate of wound healing. Vascularization was the same in the HO-1^+/+^ and HO-1^+/−^ mice, whereas in HO-1^−/−^ animals the number of vessels was significantly lower. Accordingly, overexpression of HO-1 in the HO-1^Tg^ mice was associated with strongly increased angiogenesis (Fig. 6B).

### Expression of HO-1 during wound healing in diabetic mice

**Figure 7:**
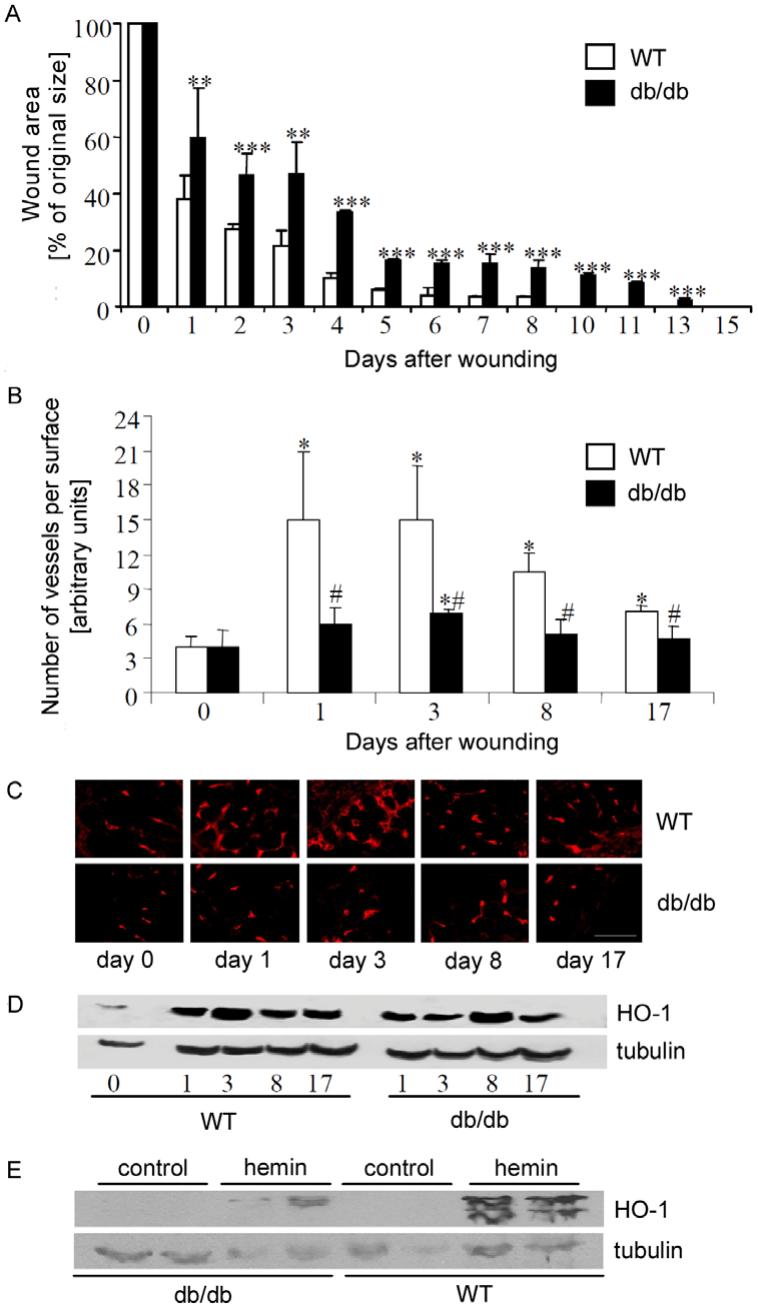
A – Closure of cutaneous wounds in the wild type (WT) and db/db diabetic C57BL mice. Each bar represents mean+SD; N = 10 animals per group. ** P<0.01, *** P<0.001 in comparison to WT animals. B – Number of vessels in healthy and wounded skin in the WT and db/db mice. Each bar represents analysis of samples from 10 animals. Data are presented as mean+SD. * P<0.05 in comparison to healthy skin (day 0); # P<0.05 in comparison to WT animals. C – Representative pictures showing blood vessels in healthy and wounded skin of WT and db/db mice. Immunohistochemical staining for CD31. Scale bar = 100 µm. D – Western blot analysis of HO-1 protein expression in healthy and wounded skin of the WT and db/db mice. One of 5 similar blots. E – Western blot analysis of HO-1 protein expression in healthy skin of the wild type and db/db mice 24 h after intradermal injection with hemin (10 mg/kg of body weight). One of 2 similar blots. Tubulin was used as a housekeeping gene to control the protein loading.

Wound healing is impaired in diabetes. We wanted to examine the expression of HO-1 in diabetic wounds. To this end, we performed experiments in hyperglycemic db/db mice, which are a model of human type 2 diabetes. As expected, db/db mice exhibited delayed wound closure compared to wild type animals (Fig. 7A). This retardation was evident throughout the whole process of healing, starting form the 1^st^ day. Wound closure in diabetic animals was completed 15 days after wounding, 5 days later than in their wild type counterparts.

Delayed healing was associated with significantly decreased tissue vascularization. In healthy skins, numbers of vessels in the wild type and db/db mice were similar, as revealed by CD31 staining. In both cases tissue injury induced angiogenesis, and vessel density reached the highest level on the 3^rd^ day. However, this increase was much weaker in diabetic mice compared to wild type animals (Fig. 7B,C). When wound healing process was ending, nonfunctional capillaries underwent regression and the number of vessels decreased in both groups.

Western blot analyses showed that expression of HO-1 was upregulated after injury both in wild type and db/db mice. However the time course of this process was different. In the wild type animals HO-1 expression reached the highest level during first three days post-wounding, whereas in db/db mice the maximal HO-1 induction was postponed, with the highest level of expression on day 8 (Fig. 7D). Thus, during the first phases of response to injury, expression of HO-1 was weaker in diabetic than in wild type animals. Such impaired induction of HO-1 we observed also after intradermal injection of hemin, a substrate and potent inducer of HO-1 expression. In wild type animals local injection of hemin resulted in a strong upregulation of HO-1 protein in the healthy skin. In contrast, the same treatment led to a very weak response in the skin of db/db mice (Fig. 7E).

### Effect of HO-1 gene transfer on wound healing in diabetic mice

Because expression of HO-1 was disturbed in the wounds of diabetic mice, we investigated whether transfer of HO-1 gene into the injured tissue may improve the healing process. To address this question, we produced adenoviral vectors harboring rat HO-1 cDNA (AdHO-1) or control GFP cDNA (AdGFP) under control of CMV promoter. *In vitro* experiments evidenced that these carriers provided high level of transgene expressions (data not shown).

**Figure 8:**
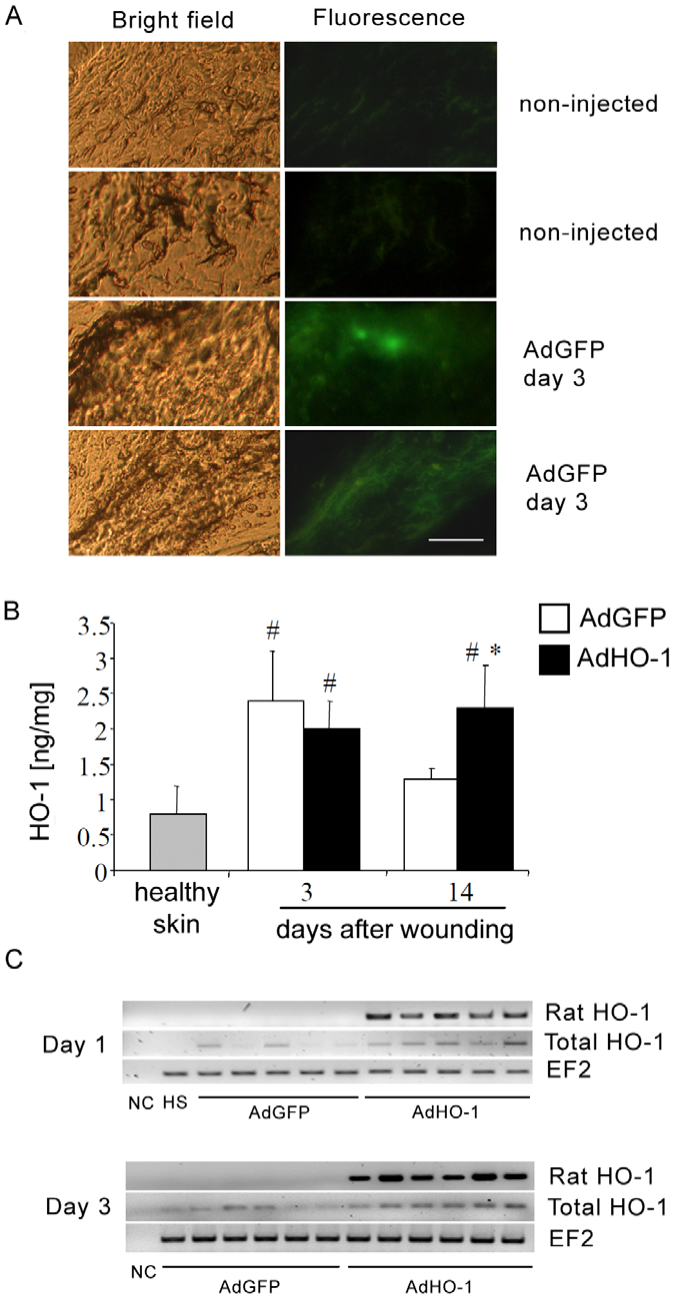
Expression of GFP and HO-1 transgenes in skin. A – Representative pictures showing the expression of GFP in the wounded skin of db/db diabetic mice, 3 days after local injection with AdGFP adenoviral vectors (2.3×10^7^ IU). Bright field and fluorescence microscopy. Scale bar = 100 µm. B – Concentration of HO-1 protein in tissue lysates of healthy and wounded skin of db/db mice, measure on the 3^rd^ and 14^th^ days after AdGFP and AdHO-1 vector delivery. Each bar represent mean+SD for 5–8 animals. # P<0.05 in comparison to healthy skin; * P<0.05 in comparison to AdGFP injected animals. C – RT-PCR analysis of rat HO-1 mRNA and total (rat/murine) mRNA in wounded skin, measured on the 1^st^ and 3^rd^ days after AdGFP and AdHO-1 vector delivery. Electrophoresis of RT-PCR products in 2% agarose gel. NC-negative control, HS-healthy skin.

Immediately after injury db/db mice were injected intradermally at four sites around the wounds either with AdGFP or AdHO-1 vectors (2.3×10^7^ IU per wound). This treatment did not induce any noticeable side effects, as evidenced by unchanged level of aspartate aminotransferase in the blood of animals (data not shown). The presence of transduced cells in the skin was confirmed by GFP expression on the 3^rd^ day in the injured tissue (Fig. 8A). We did not find GFP-positive cells in any other organ analyzed (liver, kidney, lungs, spleen), which indicates that transgene localized only near the site of injection. However, the level of transgene expression was low and we were unable to detect the differences in HO-1 protein concentration in tissue lysates prepared from the wounded skin of AdHO-1 and AdGFP injected animals on day 3 after injection (Fig. 8B). Importantly, RT-PCR with primers specific for rat HO-1 evidenced transgene expression at mRNA level already on day 1 and also on day 3 after injection (Fig. 8C). This was fully confirmed by quantitative, real-time RT-PCR (signal undetectable in AdGFP injected wounds; HO-1/EF2 ratio 0.014±0.0097 P<0.0132 for AdHO-1 treated wounds on day 1^st^). On the other hand, real-time RT-PCR with primers recognizing both murine and rat HO-1, indicated the tendency for increased expression of total HO-1 mRNA, but the differences between AdGFP and AdHO-1 injected mice did not reach statistical significance. Namely, HO-1 to EF2 reference gene ratio was 0.052±0.020 in AdGFP and 0.071±0.031 in AdHO-1 (P = 0.056) on day 1 and 0.054±0.026 in AdGFP and 0.065±0.009 (P = 0.230) on day 3. Possibly, transgene expression is masked by significant induction of endogenous enzyme in response to injury, as the HO-1/EF2 ratio in healthy skin was 0.013±0.012. It appears however, that local expression of HO-1 in the transduced cells can be upregulated. This supposition is supported by measurement of HO-1 protein at later time points, when expression of endogenous HO-1 is already diminished. Thus, although we observed similar upregulation of HO-1 expression on the 3^rd^ day post-wounding both in AdGFP and AdHO-1 treated wounds, the higher HO-1 protein concentration on 14^th^ day was observed in AdHO-1 injected wounds (Fig. 8B).

**Figure 9:**
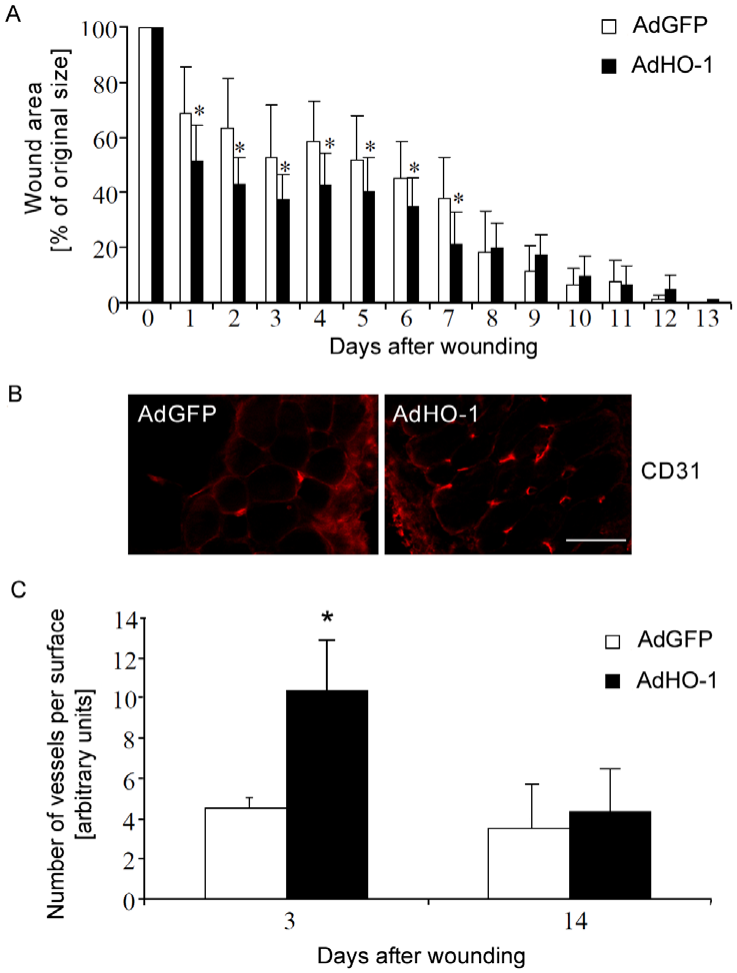
Effect of HO-1 transgene delivery on wounds. A – Effect of HO-1 transgene delivery on wound closure in the db/db diabetic mice. Adenoviral vectors (2.3×10^7^ IU in 100 µL of PBS) were injected subcutaneously near the wound immediately after injury. Control animal were injected with the same amount of AdGFP carriers. Each bar represents mean+SD; N = 5–8 animals per group. * P<0.05 in comparison to control, AdGFP treated mice. B – representative pictures showing blood vessels in the wounded skin of db/db mice injected with AdHO-1 or AdGFP vectors. CD31 staining of the skin cross-section. Scale bar = 100 µm. C – Number of vessels in wounded skin in the db/db mice injected with AdHO-1 or AdGFP, on the 3^rd^ and 14^th^ days after wounding. Analysis of specimens stained for CD31 to visualize endothelial cells. Each bar represents mean+SD values for 5–8 animals. * P<0.05 in comparison to control, AdGFP injected animals.

Despite the low level of transgene expression, wound closure analysis showed accelerated wound healing in AdHO-1 injected mice, visible during the earlier phases of healing, between days 1 and 7 after injury (Fig. 9A). Moreover, improved wound healing was accompanied by increased number of blood vessels within the wounds, as shown by staining for CD31 (Fig. 9B,C). On the 3^rd^ day, much stronger vascularization was observed in AdHO-1 injected wounds than in AdGFP treated ones. The number of capillaries decreased on 14^th^ day post-wounding. Taken together, HO-1 gene transfer may improve wound healing in db/db mice, which is associated with increased blood vessel formation.

## Discussion

HO-1 is a cytoprotective enzyme playing a role in regulation of angiogenesis and in modulation of immune response [11]–[17], [23], [24]. Its involvement in proper function of cardiovascular system, efficient neovascularization of ischemic tissues or progression of tumors is relatively well known [24]. However, significance of HO-1 in wound healing has not been established. Our study demonstrates that: i) pharmacological or genetic inhibition of HO-1 impairs healing of cutaneous wounds in mice; ii) induction of HO-1 in response to injury is impaired in diabetic mice, in which wound healing is delayed; iii) this delay may be partially reversed by HO-1 overexpression, the effect associated with increased vascularization of the wounded tissue.

Wounding leads to hemolysis and release of toxic free heme, which is a potent inducer of HO-1 expression, and a physiologic trigger to start inflammatory processes [25]. It has been suggested that HO-1 could be involved in the control of wound healing and might serve as a protective agent against oxidative and inflammatory insults [18], [25]. This suggestion has been supported by observation that pharmacological induction of HO-1 was associated with a significant acceleration of wound healing and attenuation of the inflammatory response in injured corneal epithelium [26]. Also a clinical analysis of sickle cell anemia patients, which showed a connection between a higher HO-1 activity, reflected by serum bilirubin, and protection against leg ulcers might suggest a role of HO-1 in wound healing [27].

In our model of excisional wound, the rapid upregulation of HO-1 mRNA and protein was observed upon injury (Fig. 2A), corroborating the earlier reports [18], [19]. To elucidate the role of HO-1 induction we applied two models i) pharmacological inhibition of HO-1 using SnPPIX and ii) HO-1 deficient mice. In both cases decrease in HO-1 activity impaired wound healing. In mice injected subcutaneously with SnPPIX or in HO-1 deficient individuals, the delay was statistically significant already on the first day after wounding, while in case of intraperitoneal injections of SnPPIX it became apparent on the 3^rd^ day. Probably after *s.c.* application the inhibitor was instantaneously in contact with the injured tissues while after *i.p.* delivery the saturation point of SnPPIX in the organism was reached later. It should be also kept in mind that SnPPIX, being a competitive inhibitor of HO-1, may in the same time induce HO-1 expression. Therefore it decreases enzymatic activity of HO-1, but usually is unable to block it completely. We suppose that this is a reason of much weaker effect of pharmacological than genetic inhibition of HO-1 on wound healing.

Interestingly, the effect of HO-1 deficiency increased with age and was much stronger in 6-month old than in 3-month old animals (Fig. 3A,B). Moreover, we did not observe significant delay in wound closure in younger heterozygous mice, whereas the effect of decreased level of HO-1 in older heterozygotes was strong, leading to complete inhibition of reepithelialization and formation of skin lesions in some individuals. Similar effects of aging in HO-1 deficient mice have been observed earlier in studies on endotoxemic stress [28], kidney inflammation [29], fibrosis and hepatic injury [30]. Disease symptoms typical for HO-1 deficiency, including anemia, iron-loading, lipid peroxidation, and chronic inflammation, that were not detectable at 6–9 weeks of age, became first evident by around 20 weeks [28].

Noteworthy, anemia is known to impair wound healing, as demonstrated in older people [31] or in sickle cell disease patients [27]. It is also a very frequent complication of diabetes, occurring in about 20% of patients, in whom it constitutes a significant additional burden, accompanied by systemic inflammation [32]. Increasing severity of symptoms in aging mice may suggest that effect of HO-1 deficiency on wound healing is not only direct, but may be also indirect, resulting from progressing hemolytic anemia or augmented injury of tissues caused by continues inflammatory reaction and oxidative stress.

While HO-1 inhibition impairs healing of excisional wounds, overexpression of HO-1 may facilitate this process. We demonstrated that tissue-specific upregulation of HO-1 in keratinocytes is enough to accelerate wound closure and augment vascularization. It seems important, as keratinocytes can be relatively easily isolated from patients and expanded *in vitro*. Such keratinocyte preparations are already used in clinic for treatment of burn injuries [33]. Our study may suggest that pharmacological HO-1 upregulation or gene delivery of HO-1 to the cultured cells could further improve their therapeutic potential. Indeed, as we have shown previously, HO-1 upregulates VEGF synthesis and angiogenic potential of cultured human keratinocytes [34]. The application of HO-1 by-products can be also considered, as both CO and biliverdin were demonstrated to upregulate VEGF production in endothelial cells [13] and keratinocytes [24], respectively.

Importantly, we found that expression of HO-1 can be reduced in diabetic animals. We observed impaired induction of HO-1 in response to hemin in the skin of db/db mice, when compared to the wild type counterparts. Western-blot analysis suggested also some delay in induction of HO-1 in wounded skin. Interestingly, the reduced response to hemin has been already observed in myocardium of diabetic rats [35].

Data describing effect of diabetes on HO-1 expression are, however, conflicting. It has been demonstrated, especially in chemically induced diabetes in alloxan- or streptozotocin-injected animals, that HO-1 is upregulated in different cell types, soon after induction of hyperglycemia [36]–[40]. This was possibly associated with increased oxidative stress, as HO-1 upregulation could be reduced by antioxidants [37]. However, it has been suggested that diabetes-induced oxidative stress is, in part, due to pro-oxidant activity of HO-1, mediated by increased redox-active iron [41]. Induction of HO-1 was also reported in spontaneously diabetic rats [42] and in type 2 diabetic patients, especially those with nephropathy or atherosclerosis [43]–[45].

On the contrary, there are many reports demonstrating downregulation of HO-1 expression in diabetes. Thus, the impaired levels of HO-1 were found within the ciliary bodies [46], cardiac tissue [35] or aortas [47]–[49] of streptozotocin-hyperglycemic rats and in the brain of Goto-Kakizaki rats [50]. Reduced basal expression of HO-1 was also demonstrated in type 2 diabetes patients in retinal pigment epithelium [51], leukocytes [52], and skeletal muscles [53].

Experiments carried out in animals treated with HO-1 inducers or inhibitors suggest that HO-1 may play a beneficial role in diabetes, attenuating the hyperglycemia-related complications. Thus, pharmacological activation of HO-1 in streptozotocin-injected rats led to protection of endothelial cells [47], restoration of normal rates of endothelial progenitor cell recruitment [49], and decreased production of proinflammatory cytokines after ischemia reperfusion stress [35]. This could be associated with higher concentration of serum bilirubin and reduced production of reactive oxygen species [47]. Similar finding, namely decreased oxidant production and oxidative-mediated injury was reported in patients with high HO-1 expression [44], [48].

Our data indicate for the first time that local delivery of HO-1 transgene by means of adenoviral vectors was sufficient to improve healing of excisional wounds in diabetic db/db mice, despite a low transduction efficacy. Unfortunately, we were unable to detect a difference in concentration of HO-1 protein in lysates of injured tissues between animals treated with therapeutic HO-1 or control GFP cDNAs on the 3^rd^ day after gene transfer. Possibly the expression of transgene was masked by induction of endogenous HO-1, induced by wounding, inflammatory response to adenoviral vectors and by GFP expression [54]. The ELISA assay used for measuring the HO-1 protein in tissue lysates appears to be not sensitive enough to detect a subtle difference. We demonstrated, however, that HO-1 level was sustained in AdHO-1 treated animals on day 14^th^, when endogenous HO-1 was already attenuated, which implies a successful gene delivery. Expression of GFP in control wounds and presence of rat HO-1 mRNA in AdHO-1 treated wounds, detected also on 3^rd^ day, confirms that adenoviral gene transfer was effective. It seems, that even such local overexpression of HO-1 can still improve wound closure and vascularization. Thus, a similar strategy could be proposed for diabetic patients suffering from non-healing ulcers.

There are several possible mechanisms underlying the influence of HO-1 on healing of wounds, since its products exert proangiogenic, anti-apoptotic, cytoprotective and anti-inflammatory effects. One potential pathway is an inhibition of production of inflammatory cytokines. This may facilitate the wound healing and increase the reepithelialization, as demonstrated in several reports [55]–[57]. In our model, however, the effect of HO-1 on inflammatory response is not so clear. Analysis of blood collected three days after wounding showed that concentrations of many cytokines or growth factors are higher in mice of normal level of HO-1, whereas HO-1 deficiency or inhibition results in decreased cytokine production. Such relation was observed for sCD40 antigen, IL-2, KC, IL-17, IFNγ, and GM-CSF. One can suppose, that the differences observed do not result from a direct effect of HO-1 on cytokine expressions, but are rather the consequences of different rate of wound closure, where at the same time the progression of healing is delayed in animals of lower HO-1 activity. Experiments carried out in HO-1 overexpressing mice seem to confirm lack or relatively weak influence of HO-1 on the cytokine production in response to wounding, as level of cytokines at the wound site was more dependent on wounding phase that on HO-1 activity, while production of inflammatory cytokines in transgenic keratinocytes was similar to control cells (data not shown).

It has been also postulated that HO-1 is beneficial in wound healing, due to protection of cells from toxic effects of reactive oxygen species, which are intensively generated at the site of injury, especially during first two days, in the inflammatory phase [18], [58]. Such a protective effect of HO-1 was demonstrated in many models, including ischemia/reperfusion injury [59], cigarette smoke-induced airway mucus hypersecretion [60], nephropathy [61] or corneal inflammation [26].

Significance of reduction of oxidative stress is indicated by experiments and clinical trials in which antioxidants improved wound healing, leading to increased epithelial cell proliferation and augmented angiogenesis [62]–[64]. Role of HO-1 in wounded skin may be especially important, as in cutaneous wounds the expressions of other antioxidative enzymes such as superoxide dismutase, glutathione peroxidase, glutathione-S-transferase, or catalase, as well as concentrations of ascorbic acid, vitamin D, and glutathione were significantly decreased [65].

HO-1 may also directly influence keratinocytes, facilitating their migration, improving survival in a stressful conditions of oxidative stress or hypoxia and tending to augment their proliferation. Cytoprotective and anti-apoptotic effects of HO-1 is very well documented [24]. Similarly, the important role of HO-1 in migration has been already shown in endothelial cells [13], and endothelial progenitors [17]. It seems that pro-migratory effect HO-1 depends on CO-induced phosphorylation and activation of vasodilator-activated phosphoprotein (VASP), a cytoskeletal-associated protein involved in motility, which is a downstream target for SDF-1 or IL-8 [17]. Recent study on microvascular endothelial cells has revealed that CO might regulate VASP phosphorylation and vascular cell migration also in diabetic conditions [66]. Thus, it seems that HO-1 dependent increase in migration and/or survival of keratinocytes and fibroblasts might contribute to improved wound closure.

One of the crucial processes influencing tissue regeneration is angiogenesis. Thus, disturbed blood vessel formation, for example in mice with inhibited VEGF production in epidermal keratinocytes leads to delay in wound closure [5], [67]. Several studies, including those performed in diabetic animals, have demonstrated the importance of neovascularization not only in excisional wounds [7]–[9], [17], [68], but also in burn injuries [69] or hind limb ischemia [70]. Thus, induction of angiogenesis by VEGF [4] or basic fibroblast growth factor (bFGF) [71] can be proposed as a therapeutic strategy improving wound healing. Importantly, induction of HO-1 in human keratinocytes upregulates their angiogenic potential and may restore the VEGF production impaired by high glucose level [33].

HO-1 may augment tissue neovascularization through increased production and/or activity of proangiogenic mediators, such as VEGF, monocyte chemotactic protein-1 (MCP-1), transforming growth factor-1 (TGF-β) or SDF-1, and decreased synthesis of anti-angiogenic agents, like soluble VEGF receptor 1 (sVEGFR1), soluble endoglin (sEng) or IL-10 [11], [13], [17], [24], [33]. Hence, HO-1 gene transfer to the rat ischemic muscle [15] or to pancreatic carcinoma [16] can improve angiogenesis.

As expected, we observed impaired vascularization both in HO-1 deficient mice and in db/db mice, in which induction of HO-1 upon injury was weaker. Importantly, HO-1 gene transfer, leading to faster wound closure, resulted also in significantly augmented neovascularization in diabetic animals. Similarly, overexpression of HO-1 in keratinocytes in transgenic animals was associated with increased number of blood vessels in injured tissue. This suggests that beneficial role of HO-1 in wound healing may result from its proangiogenic activity. We confirmed decreased level of VEGF in the blood of HO-1 deficient animals three days after wounding, and tendency for reduced VEGF production in the wounded skin. Additionally, overexpression of HO-1 in keratinocytes was associated with slight, but statistically significant increase in VEGF synthesis in hypoxia. Stimulatory effect of HO-1 was also reported for hypoxia-induced VEGF expression in human keratinocytes [33]. Interestingly, in mice injected with SnPPIX, we did not observe decreased expression of VEGF in the blood (Table 1). Moreover, there was no difference in expression of VEGF in wounded skin and in number of blood vessels (data not shown). This may suggest that many effects of SnPPIX are HO-1 independent and that inhibition of wound closure by SnPPIX and by HO-1 deficiency relies on distinct mechanisms. Similar observations were reported in studies on effects of pharmacological or genetic HO-1 inhibition in cancer progression [72].

We found the reduced concentration of soluble CD40 antigen and IL-17 in HO-1 deficient mice. Both proteins may stimulate angiogenesis [73], [74]. Similar decrease was observed for proangiogenic IL-2 and KC, the cytokines which can accelerate the wound healing process through stimulating the proliferation of dermal fibroblasts or through augmenting the maturation of granulose tissue, respectively [75], [76]. Finally low activity of HO-1 led to decreased level of GM-CSF, the proangiogenic growth factor which facilitates wound contraction and induces keratinocyte proliferation and migration [77]. One could suggest that reduction of those mediators might contribute to wound healing impairment observed in HO-1 deficient mice.

In summary, our study indicates that activation of HO-1 accelerates wound healing in normoglycemic and diabetic mice, what can be associated with augmented angiogenesis, increased migratory capacities and improved survival of keratinocytes. Overexpression of HO-1 by *in vivo* gene transfer or *ex vivo* transduction of keratinocytes may be proposed as a strategy for improvement of wound healing in diabetic patients.

## Materials and Methods

### Ethics statement

All animals were handled in strict accordance with good animal practice as defined by the relevant national and/or local animal welfare bodies, and all animal work was approved by the Local Ethical Committee for Animal Research at the Jagiellonian University.

### Reagents

SnPPIX was from Frontier Scientific (Carnforth, UK); Mouse Cytokine Antibody Array kit from Ray-Biotech (Norcross, GA); Anti-mouse CD31 and rhodamine-conjugated rat anti-mouse antibodies from BD Biosciences (Franklin Lakes, NJ); Rabbit anti-HO-1 antibody from Stressgen (Ann Arbor, MI); goat anti-rabbit-HRP antibody from CellSignaling Technology (Danvers, MA); Dispase, trypsin-EDTA, glutamine, sodium pyruvate, nonessential amino acids, penicillin, streptomycin, β-mercaptoethanol, DMEM, and FCS from Gibco (Carlsbad, CA); Epidermal Keratinocyte Medium from CELLnTEC (Bern, Switzerland); Kit for ASPAT measurement from Biomerieux (Marcy l'Etoile, France); ELISA for VEGF from R&D (Minneapolis, MN); QIAzol from Qiagen (Valencia, CA); Turbo DNA Free Kit from Ambion (Austin, TX); M-MLV and oligo(dT)_15_ primers from Promega (Madison, WI); DNAzyme-II DNA polymerase from Finnzymes (Espoo, Finland); Cell Proliferation Kit from Roche (Basel, Switzerland); Adeno-X Virus Production Kit, Adeno-X Virus Purification Kit, and Adeno-X rapid titer ELISA from Clontech (Mountain View, CA); All other reagents were from Sigma-Aldrich (St. Louis, MO). All materials were approved for investigational-use only.

### Cell culture

HaCaT cells (immortalised human keratinocytes), kindly provided by Dr. Robert Fusening (Heidelberg University, Germany), were grown in DMEM medium, supplemented with FBS (10%), penicillin (100 U/ml) and streptomycin (10 µg/ml).

### Isolation and culture of primary keratinocytes

Skins were excised from 1-5-day old pups, purified of fat, washed in PBS with penicillin (100 U/ml) and streptomycin (100 µg/ml), and incubated overnight with dispase (6 U/ml). Epidermis was removed with forceps, placed in 0.05% trypsin-EDTA solution for a few minutes, and neutralized using complete DMEM with 10% FCS. Cell suspension was centrifuged at 200 *g* for 5 minutes at room temperature. The isolated keratinocytes were cultured in serum free CnT-07 PCT Epidermal Keratinocyte Medium.

### Detection of human and rat HO-1 mRNA in the skin

RNA was extracted from homogenized healthy or wounded skins using QIAzol and purified with Turbo DNA Free^TM^ kit. Two micrograms of total RNA was used for reverse transcription with M-MLV and oligo(dT) primers. PCR was performed with primers for human HO-1 (F: 5′-GGAGGTCATCCCCTACACACC-3′; R: 5′-CTGGGAGCGGGTGTTGAGTG-3′), and constitutive human EF2 (F: 5′-GACATCACCAAGGGTGTGCAG-3′; R: 5′-TCAGCACACTGGCATAGAGGC-3′) or with primers for rat HO-1 (F: 5′-AGAGTCCCTCACAGACAGAGTTT-3′; R: 5′-CCTGCAGAGAGAAGGCTACATGA-3′), murine/rat HO-1 (F: 5′-CTTTCAGAAGGGYCAGGTGWCC-3′; R: 5′-GTGGAGMCGCTTYACRTAGYGC-3′) and constitutive murine EF2 (F: 5′GACATCACCAAGGGTGTGCAG-3′; R: 5′-TCAGCACACTGGCATAGAGGC-3′). Real-time PCR reaction was done in cycles: 95°C for 30 s, 60°C for 60 s, 72°C for 45 s; data were analyzed with ΔCt method. In regular PCR the reaction was done in cycles: 95°C for 45 s, 60°C for 30 s, 72°C for 60 s. We did 25 cycles for total (murine/rat) HO-1, and 35 cycles for rat HO-1.

### Cell proliferation assay

Spontaneous cell proliferation was assessed by BrdU incorporation colorimetric assay performed according to the manufacturer's protocol. Cells (5,000 per well) were seeded in 96-well plate and assayed 48 h later.

### Migration assay

Cells were grown to full confluence and treated with 10 mM hydroxyurea to block proliferation. A scratch was generated with a pipette tip. Photographs were taken at different time points, always from the same place, and analyzed using ImageJ programme (National Institute of Health, http://rsb.info.nih.gov/ij).

### MTT reduction assay

Thiazolyl blue tetrazolium bromide solution was added to the cells (0.5 mg/ml) for 0.5–1 h. Then, medium was removed and 100 µl of 2-propanol with 5 mM HCl were added to dissolve the formazan crystals. Optical density was measured at 560 nm.

### Preparation of adenoviral vectors

Adenoviral vectors containing rat HO-1 cDNA (Ad-HO1) were kindly gifted by Dr. Gisa Tiegs (Erlangen, Germany). Control vectors harboring GFP cDNA (Ad-GFP) were produced using the Adeno-X system. Both vectors were propagated in 293 cells, purified using Adeno-X Virus Purification kit, and titrated with Adeno-X rapid titer ELISA kit.

### Animals and wound healing model

Mice were anesthetized with isoflurane, wiped with 70% ethanol and shaved. Once the skin was exposed, two full-thickness (including panniculus carnosus) circular wounds (4 mm in diameter) on each animal were created using disposable biopsy punch. Each wound was photographed every day and analyzed using ImageJ software. For biochemical or immunohistochemical analysis wounded skin, together with a margin of healthy skin, was excised using 8 mm-diameter biopsy punch.

#### Injection with SnPPIX

Immediately after wounding, mice (C57Bl/6, 3-month old) were divided into four groups and injected daily either intraperitoneally or subcutaneously with SnPPIX (45 µmol/kg of body weight, dissolved in 0.2 M NaOH), or with a vehicle (0.2 M NaOH in PBS).

#### Adenoviral-mediated HO-1 gene transfer

Mice (db/db, 3-month old) were injured and injected intracutaneously at 3 points near the wound with AdGFP or AdHO-1 (100 µl, 2.3×10^8^ IU/ml per wound). Each mouse received the same vector/vehicle in both wounds to avoid possible effects from leakage.

#### HO-1 induction in skin in mice after injection with hemin

C57BL wild type and C57BL db/db mice were injected intradermally with hemin (10 mg/kg of body weight). Animals were sacrificed after 24 hours and HO-1 expression was determined by immunoblotting.

### Protein isolation and western blotting

Skin was homogenized, lysed in 200 µl of ice-cold lysis buffer (PBS with 1% Triton X-100, 0.1 µg/ml PMSF, 1 µg/ml leupeptin and 1 µg/ml aprotinin), incubated on ice for 30 minutes, and centrifuged at 10,000 rpm at 4°C for 10 min. Supernatants were stored at −80°C. Samples were subjected to SDS-PAGE and immunoblotting as described earlier [13].

### CD31 staining

The wounds were cut into 8 µm slides. Cryosections were dried and blocked for at least 1 h with 10% goat serum, 0.05% Tween and 0.1% Triton ×100 in PBS. Either rat anti-mouse CD31 or isotypic antibodies 1∶100 in 10× diluted blocking buffer were applied for 1.5 h and then sections were washed with PBS. Secondary antibodies conjugated with rhodamine 1∶1000 in PBS were applied for 0.5 h and the sections were washed with PBS. Slides were analyzed under the fluorescent microscope. Capillaries were counted in panniculus carnosus layer, in the direct vicinity of granulation tissue, at 400× magnification.

### Mouse cytokine antibody array

Cytokines in blood serum were analyzed using Ray-Biotech Mouse Cytokine Antibody Array kit, according to vendor's protocol.

### Statistical analysis

Statistical analysis was done using Student's t test or ANOVA followed by Tukey test. Data are presented as a mean±SD of 3–6 experiments.

## References

[1] Gurtner GC, Werner S, Barrandon Y, Longaker MT (2008). Wound repair and regeneration.. Nature.

[2] Stadelmann WK, Digenis AG, Tobin GR (1998). Physiology and healing dynamics of chronic cutaneous wounds.. Am J Surg.

[3] Falanga V (2005). Wound healing and its impairment in the diabetic foot.. Lancet.

[4] Michaels J, Dobryansky M, Galiano RD, Bhatt KA, Ashinoff R (2005). Topical vascular endothelial growth factor reverses delayed wound healing secondary to angiogenesis inhibitor administration.. Wound Repair Regen.

[5] Bloch W, Huggel K, Sasaki T, Grose R, Bugnon P (2000). The angiogenesis inhibitor endostatin impairs blood vessel maturation during wound healing.. Faseb J.

[6] Howdieshell TR, Callaway D, Webb WL, Gaines MD, Procter CD (2001). Antibody neutralization of vascular endothelial growth factor inhibits wound granulation tissue formation.. J Surg Res.

[7] Dickens S, Vermeulen P, Hendrickx B, Van den Berge S, Vranckx JJ (2008). Regulable vascular endothelial growth factor165 overexpression by ex vivo expanded keratinocyte cultures promotes matrix formation, angiogenesis, and healing in porcine full-thickness wounds.. Tissue Eng Part A.

[8] Galiano RD, Tepper OM, Pelo CR, Bhatt KA, Callaghan M (2004). Topical vascular endothelial growth factor accelerates diabetic wound healing through increased angiogenesis and by mobilizing and recruiting bone marrow-derived cells.. Am J Pathol.

[9] Suh W, Kim KL, Kim JM, Shin IS, Lee YS (2005). Transplantation of endothelial progenitor cells accelerates dermal wound healing with increased recruitment of monocytes/macrophages and neovascularization.. Stem Cells.

[10] Maines MD (1997). The heme oxygenase system: a regulator of second messenger gases.. Annu Rev Pharmacol Toxicol.

[11] Dulak J, Jozkowicz A, Foresti R, Kasza A, Frick M (2002). Heme oxygenase activity modulates vascular endothelial growth factor synthesis in vascular smooth muscle cells.. Antioxid Redox Signal.

[12] Jozkowicz A, Huk I, Nigisch A, Weigel G, Weidinger F (2002). Effect of prostaglandin-J(2) on VEGF synthesis depends on the induction of heme oxygenase-1.. Antioxid Redox Signal.

[13] Jozkowicz A, Huk I, Nigisch A, Weigel G, Dietrich W (2003). Heme oxygenase and angiogenic activity of endothelial cells: stimulation by carbon monoxide and inhibition by tin protoporphyrin-IX.. Antioxid Redox Signal.

[14] Cisowski J, Loboda A, Jozkowicz A, Chen S, Agarwal A (2005). Role of heme oxygenase-1 in hydrogen peroxide-induced VEGF synthesis: effect of HO-1 knockout.. Biochem Biophys Res Commun.

[15] Suzuki M, Iso-o N, Takeshita S, Tsukamoto K, Mori I (2003). Facilitated angiogenesis induced by heme oxygenase-1 gene transfer in a rat model of hindlimb ischemia.. Biochem Biophys Res Commun.

[16] Sunamura M, Duda DG, Ghattas MH, Lozonschi L, Motoi F (2003). Heme oxygenase-1 accelerates tumor angiogenesis of human pancreatic cancer.. Angiogenesis.

[17] Deshane J, Chen S, Caballero S, Grochot-Przeczek A, Was H (2007). Stromal cell-derived factor 1 promotes angiogenesis via a heme oxygenase 1-dependent mechanism.. J Exp Med.

[18] Hanselmann C, Mauch C, Werner S (2001). Haem oxygenase-1: a novel player in cutaneous wound repair and psoriasis?. Biochem J.

[19] Kampfer H, Kolb N, Manderscheid M, Wetzler C, Pfeilschifter J (2001). Macrophage-derived heme-oxygenase-1: expression, regulation, and possible functions in skin repair.. Mol Med.

[20] Platt JL, Nath KA (1998). Heme oxygenase: protective gene or Trojan horse.. Nat Med.

[21] Jozkowicz A, Dulak J (2003). Effects of protoporphyrins on production of nitric oxide and expression of vascular endothelial growth factor in vascular smooth muscle cells and macrophages.. Acta Biochim Pol.

[22] Grundemar L, Ny L (1997). Pitfalls using metalloporphyrins in carbon monoxide research.. Trends Pharmacol Sci.

[23] Maines MD (1988). Heme oxygenase: function, multiplicity, regulatory mechanisms, and clinical applications.. Faseb J.

[24] Loboda A, Jazwa A, Grochot-Przeczek A, Rutkowski A, Cisowski J (2008). : Heme Oxygenase-1 and the Vascular Bed: From Molecular Mechanisms to Therapeutic Opportunities.. Antioxid Redox Signal.

[25] Wagener FA, van Beurden HE, von den Hoff JW, Adema GJ, Figdor CG (2003). The heme-heme oxygenase system: a molecular switch in wound healing.. Blood.

[26] Patil K, Bellner L, Cullaro G, Gotlinger KH, Dunn MW (2008). Heme oxygenase-1 induction attenuates corneal inflammation and accelerates wound healing after epithelial injury.. Invest Ophthalmol Vis Sci.

[27] Nolan VG, Adewoye A, Baldwin C, Wang L, Ma Q (1997). Sickle cell leg ulcers: associations with haemolysis and SNPs in Klotho, TEK and genes of the TGF-beta/BMP pathway.. Br J Haematol.

[28] Poss KD, Tonegawa S (1997). Heme oxygenase 1 is required for mammalian iron reutilization.. Proc Natl Acad Sci U S A.

[29] Pittock ST, Norby SM, Grande JP, Croatt AJ, Bren GD (2005). MCP-1 is upregulated in unstressed and stressed HO-1 knockout mice: Pathophysiologic correlates.. Kidney Int.

[30] Poss KD, Tonegawa S (1997). Reduced stress defense in heme oxygenase 1-deficient cells.. Proc Natl Acad Sci U S A.

[31] Frank C (2004). Approach to skin ulcers in older patients.. Can Fam Physician.

[32] Thomas M, Tsalamandris C, MacIsaac R, Jerums G (2005). Anaemia in diabetes: an emerging complication of microvascular disease.. Curr Diabetes Rev.

[33] Drukala J, Bandura L, Cieslik K, Korohoda W (2001). Locomotion of human skin keratinocytes on polystyrene, fibrin, and collagen substrata and its modification by cell-to-cell contacts.. Cell Transplantation.

[34] Jazwa A, Loboda A, Golda S, Cisowski J, Szelag M (2006). Effect of heme oxygenase-1 on VEGF synthesis and angiogenic potency of human keratinocytes.. Free Radical Biol Med.

[35] Di Filippo C, Marfella R, Cuzzocrea S, Piegari E, Petronella P (2005). Hyperglycemia in streptozotocin-induced diabetic rat increases infarct size associated with low levels of myocardial HO-1 during ischemia/reperfusion.. Diabetes.

[36] Hayashi K, Haneda M, Koya D, Maeda S, Isshiki K (2001). Enhancement of glomerular heme oxygenase-1 expression in diabetic rats.. Diabetes Res Clin Pract.

[37] Koya D, Hayashi K, Kitada M, Kashiwagi A, Kikkawa R (2003). Effects of antioxidants in diabetes-induced oxidative stress in the glomeruli of diabetic rats.. J Am Soc Nephrol.

[38] Cukiernik M, Mukherjee S, Downey D, Chakabarti S (2003). Heme oxygenase in the retina in diabetes.. Curr Eye Res.

[39] Song F, Qi X, Chen W, Jia W, Yao P (2007). Effect of Momordica grosvenori on oxidative stress pathways in renal mitochondria of normal and alloxan-induced diabetic mice. Involvement of heme oxygenase-1.. Eur J Nutr.

[40] Oksala NK, Lappalainen J, Laaksonen DE, Khanna S, Kaarniranta K (2007). Alpha-lipoic acid modulates heat shock factor-1 expression in streptozotocin-induced diabetic rat kidney.. Antioxid Redox Signal.

[41] Farhangkhoee H, Khan ZA, Mukherjee S, Cukiernik M, Barbin YP (2003). Heme oxygenase in diabetes-induced oxidative stress in the heart.. J Mol Cell Cardiol.

[42] Cosso L, Maineri EP, Traverso N, Rosatto N, Pronzato MA (2001). Induction of heme oxygenase 1 in liver of spontaneously diabetic rats.. Free Radic Res.

[43] Avogaro A, Pagnin E, Calo L (2003). Monocyte NADPH oxidase subunit p22(phox) and inducible hemeoxygenase-1 gene expressions are increased in type II diabetic patients: relationship with oxidative stress.. J Clin Endocrinol Metab.

[44] Calabrese V, Mancuso C, Sapienza M, Puleo E, Calafato S (2007). Oxidative stress and cellular stress response in diabetic nephropathy.. Cell Stress Chaperones.

[45] Song J, Sumiyoshi S, Nakashima Y, Doi Y, Ida M (2009). Overexpression of heme oxygenase-1 in coronary atherosclerosis of Japanese autopsies with diabetes mellitus: Hisayama study.. Atherosclerosis.

[46] Rossi S, D'Amico M, Capuano A, Romano M, Petronella P (2006). Hyperglycemia in streptozotocin-induced diabetes leads to persistent inflammation and tissue damage following uveitis due to reduced levels of ciliary body heme oxygenase-1.. Mediators Inflamm.

[47] Quan S, Kaminski PM, Yang L, Morita T, Inaba M (2004). Heme oxygenase-1 prevents superoxide anion-associated endothelial cell sloughing in diabetic rats.. Biochem Biophys Res Commun.

[48] Abraham NG, Rezzani R, Rodella L, Kruger A, Taller D (2004). Overexpression of human heme oxygenase-1 attenuates endothelial cell sloughing in experimental diabetes.. Am J Physiol Heart Circ Physiol.

[49] Sambuceti G, Morbelli S, Vanella L, Kusmic C, Marini C (2009). Diabetes impairs the vascular recruitment of normal stem cells by oxidant damage; Reversed by increases in pAMPK, heme oxygenase-1 and adiponectin.. Stem Cells.

[50] Moreira TJ, Cebere A, Cebers G, Ostenson CG, Efendic S (2007). Reduced HO-1 protein expression is associated with more severe neurodegeneration after transient ischemia induced by cortical compression in diabetic Goto-Kakizaki rats.. J Cereb Blood Flow Metab.

[51] Da Silva JL, Stoltz RA, Dunn MW, Abraham NG, Shibahara S (1997). Diminished heme oxygenase-1 mRNA expression in RPE cells from diabetic donors as quantitated by competitive RT/PCR.. Curr Eye Res.

[52] Adaikalakoteswari A, Balasubramanyam M, Rema M, Mohan V (2006). Differential gene expression of NADPH oxidase (p22phox) and hemoxygenase-1 in patients with type 2 diabetes and microangiopathy.. Diabet Med.

[53] Bruce CR, Carey AL, Hawley JA, Febbraio MA (2003). Intramuscular heat shock protein 72 and heme oxygenase-1 mRNA are reduced in patients with type 2 diabetes: evidence that insulin resistance is associated with a disturbed antioxidant defense mechanism.. Diabetes.

[54] Zhang F, Hackett NR, Lam G, Cheng J, Pergolizzi R (2003). Green fluorescent protein selectively induces HSP70-mediated upregulation of COX-2 expression in endothelial cells.. Blood.

[55] Ashcroft GS, Mills SJ (2002). Androgen receptor-mediated inhibition of cutaneous wound healing.. J Clin Invest.

[56] Gilliver SC, Ashworth JJ, Mills SJ, Hardman MJ, Ashcroft GS (2006). Androgens modulate the inflammatory response during acute wound healing.. J Cell Sci.

[57] Goren I, Müller E, Schiefelbein D, Christen U, Pfeilschifter J (2007). Systemic anti-TNFα treatment restores diabetes-impaired skin repair in ob/ob mice by inactivation of macrophages.. J Invest Dermatol.

[58] Ojha N, Roy S, He G, Biswas S, Velayutham M (2008). Assessment of wound-site redox environment and the significance of Rac2 in cutaneous healing.. Free Radic Biol Med.

[59] Dulak J, Deshane J, Jozkowicz A, Agarwal A (2008). Heme oxygenase-1 and carbon monoxide in vascular pathobiology. Focus on angiogenesis.. Circulation.

[60] Almolki A, Guenegou A, Golda S, Boyer L, Benallaoua M (2008). Heme oxygenase-1 prevents airway mucus hypersecretion induced by cigarette smoke in rodents and humans.. Am J Pathol.

[61] Wu CC, Lu KC, Chen JS, Hsieh HY, Lin SH (2008). HO-1 induction ameliorates experimental murine membranous nephropathy: anti-oxidative, anti-apoptotic and immunomodulatory effects.. Nephrol Dial Transplant.

[62] Altavilla D, Galeano M, Bitto A, Minutoli L, Squadrito G (2005). Lipid peroxidation by raxofelast improves angiogenesis and wound healing in experimental burn wounds.. Shock.

[63] Altavilla D, Saitta A, Cucinotta D, Galeano M, Deodato B (2001). Inhibition of lipid peroxidation restores impaired vascular endothelial growth factor expression and stimulates wound healing and angiogenesis in the genetically diabetic mouse.. Diabetes.

[64] Theilla M, Singer P, Cohen J, Dekeyser F (2007). A diet enriched in eicosapentanoic acid, gamma-linolenic acid and antioxidants in the prevention of new pressure ulcer formation incritically ill patients with acute lung injury: a randomize, prospective, controlled study.. Clin Nutr.

[65] Shukla A, Rasik AM, Patnaik GK (1997). Depletion of reduced glutathione, ascorbic acid, vitamin E and antioxidant defence enzymes in a healing cutaneous wound.. Free Radic Res.

[66] Li Calzi S, Purich DL, Chang KH, Afzal A, Nakagawa T (2008). Carbon Monoxide and Nitric Oxide Mediate Cytoskeletal Reorganization in Microvascular Cells via Vasodilator-Stimulated Phosphoprotein (VASP) Phosphorylation: Evidence for Blunted Responsiveness in Diabetes.. Diabetes.

[67] Rossiter H, Barresi C, Pammer J, Rendl M, Haigh J (2004). Loss of vascular endothelial growth factor activity in murine epidermal keratinocytes delays wound healing and inhibits tumor formation.. Cancer Res.

[68] Galeano M, Deodato B, Altavilla D, Cucinotta D, Arsic N (2003). Adeno-associated viral vector-mediated human vascular endothelial growth factor gene transfer stimulates angiogenesis and wound healing in the genetically diabetic mouse.. Diabetologia.

[69] Galeano M, Deodato B, Altavilla D, Squadrito G, Seminara P (2003). Effect of recombinant adeno-associated virus vector-mediated vascular endothelial growth factor gene transfer on wound healing after burn injury.. Crit Care Med.

[70] Ebrahimian TG, Heymes C, You D, Blanc-Brude O, Mees B (2006). NADPH oxidase-derived overproduction of reactive oxygen species impairs postischemic neovascularization in mice with type 1 diabetes.. Am J Pathol.

[71] Zhao W, Han Q, Lin H, Gao Y, Sun W (2008). Improved neovascularization and wound repair by targeting human basic fibroblast growth factor (bFGF) to fibrin.. J Mol Med.

[72] Jozkowicz A, Was H, Dulak J (2007). Heme oxygenase-1 in tumor: is it a false friend?. Antioxid Redox Signal.

[73] Minuzzo S, Moserle L, Indraccolo S, Amadori A (2007). Angiogenesis meets immunology: cytokine gene therapy of cancer.. Mol Aspects Med.

[74] Honorati MC, Neri S, Cattini L, Facchini A (2006). Interleukin-17, a regulator of angiogenic factor release by synovial fibroblasts.. Osteoarthritis Cartilage.

[75] Singh H, Abdullah A, Herndon DN (1992). Effects of rat interleukin-2 and rat interferon on the natural killer cell activity of rat spleen cells after thermal injury.. J Burn Care Rehabil.

[76] Moyer KE, Saggers GC, Allison GM, Mackay DR, Ehrlich HP (2002). Effects of interleukin-8 on granulation tissue maturation.. J Cell Physiol.

[77] Groves RW, Schmidt-Lucke JA (2000). Recombinant human GM-CSF in the treatment of poorly healing wounds.. Adv Skin Wound Care.

